# Early Adulthood and Care Giving Professions in the 21st Century: Mental Wellness as an Institutional Priority and Reward Strategy

**DOI:** 10.1177/00469580251399302

**Published:** 2025-12-01

**Authors:** Linda S. Pagani, Nairy Kazandjian, Steve Geoffrion, Tracie A. Barnett

**Affiliations:** 1 Université de Montréal, QC, Canada; 2 McGill University, Montreal, QC, Canada

**Keywords:** millennial recruitment, millennial attrition, mental health, well-being, mental literacy

## Abstract

Globally, health care is presently characterized by profound recruitment/retention difficulties. Its systems are currently experiencing an unprecedented workforce crisis marked by high attrition rates and mental health challenges from recruitment onward. This is especially urgent in professions that risk burnout, such as those that expose workers to secondary trauma and require societally undervalued compassionate labor skills (eg, empathy, meticulousness, patience). While numerous studies have highlighted these risks, fewer have explored how institutional structures can proactively respond to this generational shift. Our goal is to provide integrative position paper that synthesizes key findings on etiological and concomitant factors into a structured framework inspired by self-determination and subjective well-being theories to inform practice, policy, and future research. We integrate scientific evidence to examine key factors behind psychological distress in two-thirds of university students and health care recruits in mostly female professions. Psychological difficulties have associated risks for recruitment/performance/retention of workers. Specific pre-existing individual characteristics must be considered, especially in incoming recruits of this generation. This synthesis proposes mental wellness as a central strategy for recruitment and retention in human resource management. Increased and pre-esisting distress affects worker retention. Individual vulnerabilities such as parenting, smartphone and social media use, loneliness, and pre-existing conditions play a role. Consequently, educational and health care institutions should prioritize strategies that enhance subjective well-being, transparency, and work-life balance. Psychological training focused on self-awareness, character strengths, stress management, and growth-oriented effort-reward dynamics is essential for retaining young health care professionals and ensuring workforce, workplace, and worker sustainability.

**What do we already know about this topic?**
● Globally, more than half of university students screen positive functional impairment related to major depression, mania/hypomania, generalized anxiety disorder, panic disorder, alcohol use disorder, and substance use disorder during the past year. Employers of university-educated healthcare workers must not expect such mental health risks to disappear upon graduation.
**How does your research contribute to the field?**
● We provide strategies to protect well-being and prevent disease. In health care, secondary trauma and burnout syndrome, both closely linked to compassion fatigue, significantly impact professional performance and retention of workers at all levels. Educational and healthcare institutions should integrate these values into their recruitment and retention policies to better meet the needs of new recruits.

## Introduction

Health and educational systems are currently experiencing an unprecedented workforce crisis marked by high attrition rates and mental health challenges from recruitment onward.^1^ This is especially urgent in caregiving professions, where compassionate labor skills and risks for secondary trauma contribute to rising attrition and burnout.^2^ While numerous studies have highlighted these risks, fewer have commented on how institutional structures can proactively respond to this generational shift.

**Figure 1.**
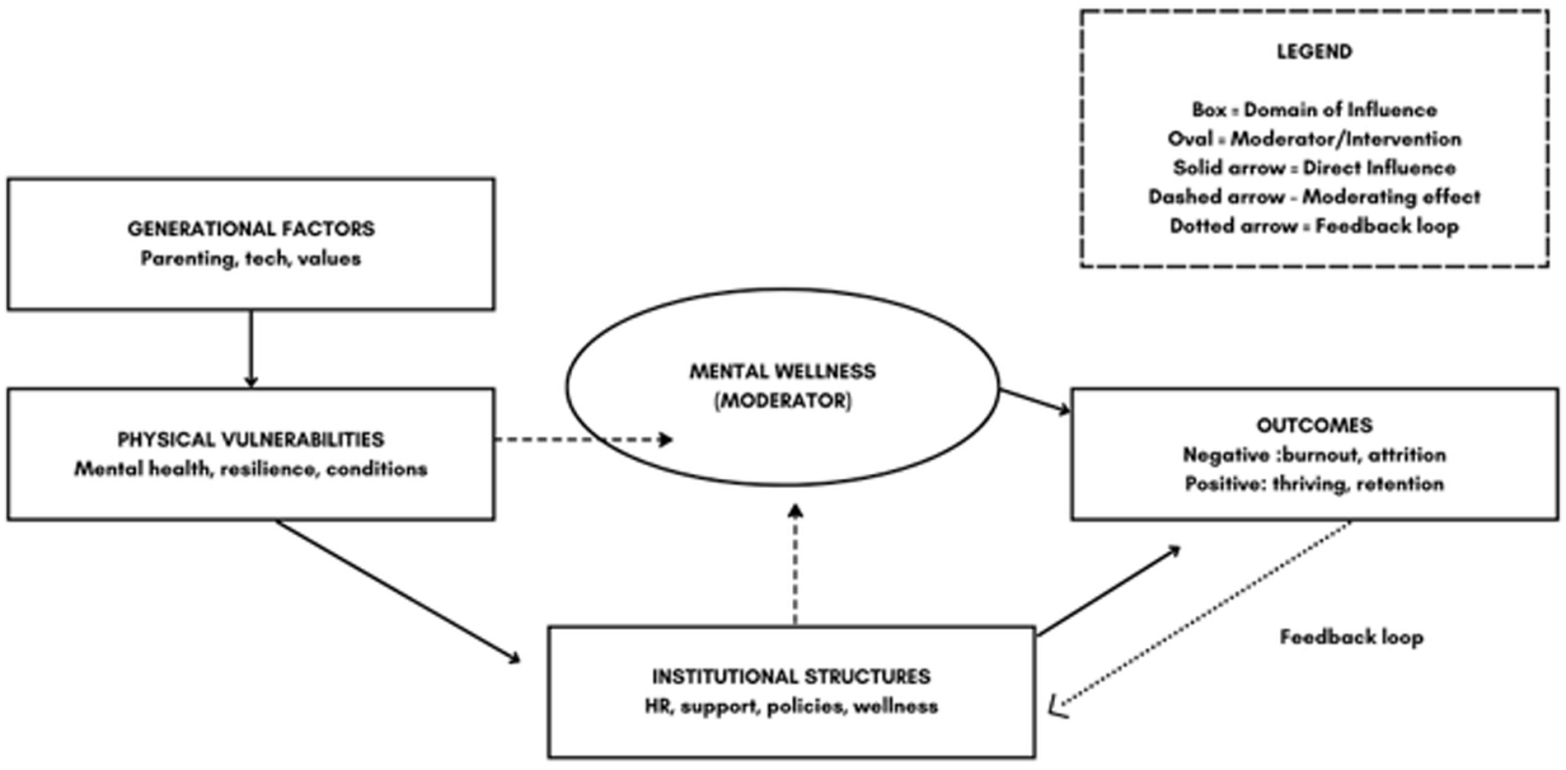
A conceptual model illustrating the connection between generational factors, psychological vulnerabilities, institutional structures, and retention outcomes.

This integrative position paper offers a “viewpoint of the literature,” as conceptualized by Grant and Booth,^3^ to reimagine mental wellness as a strategic lever for recruitment and retention. Drawing on high-quality clinical evidence from the past decade, we synthesize key insights into the psychosocial mechanisms such as early parenting exposures, smartphone dependence, social media use, loneliness, and pre-existing mental health condition. These contribute to subjective distress in emerging adulthood.^4^ Through a curated review of the most relevant literature on university students and early-career recruits in the healthcare sector, we develop a structured framework that informs practice, policy, and future research. Our aim is to position mental wellness not as a reactive concern, but as a proactive foundation for sustaining workforce engagement across all levels. Figure 1 offers a conceptual model illustrating the connection between generational factors, psychological vulnerabilities, institutional structures, and retention outcomes.

Educational and health institutions face a new generation of young adults with distinct strengths and vulnerabilities.^4,5^ A persistent rise in mental health issues among emerging adults has become a dominant societal challenge,^6^ with long-term projected global repercussions including weaker GDPs, solitude, and dwindling birthrates.^7^

Workplaces must recognize traits beyond achievement, such as character strengths and personality profiles, as vital to personal well-being and success.^8^ Yet, these “soft” skills are often overlooked in recruitment. That is, institutions tend to overvalue cognitive characteristics and undervalue societally compassionate labor characteristics (eg, empathy, meticulousness, patience) which remain core features in health care.^9,10^ To foster flourishing, labor markets need innovative approaches to balance both features.^11^ In health care, this directly affects recruit retention and care quality.^12^

## Generational Differences and Technology

Young adults born since the mid-1990s now comprise the largest generational cohort in education and the workforce.^13,14^ They exhibit distinct values and behaviors across various demographic indicators,^15^ including technological adaptability, confidence, and diversity.^16^ Compared to earlier generations, they favor job mobility, professional growth, and open, feedback-oriented leadership,^5,15^ as well as civic engagement and autonomy.^17^ Their work preferences center on collaboration, flexibility, personal development, and respect for non-work time, with expectations for shared, purpose-driven leadership.^18^

Health data from the UK shows tripling attrition rates and declining satisfaction with leisure time among this demographic.^12^ Work-life imbalance cited as a major cause of professional attrition and burnout.^19^ Educational and labor institutions led by older generations must recognize current generational differences to build a sustainable workforce.^20^ However, many still rely on outdated values and rigid structures, overlooking vital psychological and social factors unless externally mandated.^21^ This risks workplace misalignment for young recruits in service-based professions.^15^

## Mental Health Challenges in Young Adults

Regardless of cultural setting, young adulthood is a critical period of biological and psychological transition, marked by identity development challenges, career entry, new relationships, and increasing societal expectations for autonomy and responsibility.^17^ Such opportunities and demands overlap with developmental psychopathological risk.^6,22^ Noteworthy is that the peak prevalences of both psychiatric illness^23^ and loneliness^20^ are between ages 18 and 25.

Spanning the 1990s through 2016, rates of mental health disorders among young and emerging adults have tripled worldwide.^24^ Regardless of education status, up to 20% of emerging adults have a mental disorder worldwide, with another 20% reporting undiagnosed yet impairing mental distress at varying degrees across the mental health continuum.^23,25^ The increased populational prevalence and developmental persistence of autism spectrum disorder (at an upper limit of 2.5%), Attention Deficit Hyperactivity Disorder (at an upper limit of 16%), and Learning Disabilities (at an upper limit of 12%) add an additional population health burden on the workforce.^6,26,27^ The rates of pharmacotherapy have also been on the rise around the globe.^23,28,29^ Although pharmacotherapy can reduce negative emotion, this reduction does not necessarily lead to an improved understanding of mental health disorders and strategies for maintaining well-being.^30^ Consequently, several elephants in the room need to be addressed when speaking of the recently born.

## Factors Contributing to Mental Health Challenges in Young Adults

The rising prevalence of psychiatric disorders has significantly impacted universities and colleges.^31-35^ Global research finds that between one- and two-thirds of first-year students screen positive for a lifetime mental disorder, while 20% experience functional impairment.^23,28,29,36,37^ Depressive symptoms and frequent substance use behaviors are most common, with onset typically before college. More recently, in a study of first year students across 18 countries,^22^ Mason et al^22^ mapped age-of-onset and course of disorders and observed that many difficulties begin before or during the first year of university. Two-thirds of first-year university students screened positive for lifetime mental disorders and more than half screened positive for 12-month mental disorders. Specifically: 29.5% of students reported moderate to severe depressive symptoms; 39% experiencing moderate, severe, or extremely severe anxiety symptoms; Higher rates occurred among females and younger students. Males reported higher substance and attention-deficit/hyperactivity disorders. 21.3% reported having experienced suicidal thoughts at some point in their lives; and 12.1% had experienced suicidal thoughts in the past year. Although prevalence rates varied by country, the overall burden of mental health disorders was consistently high. Alarmingly, help-seeking behavior was often low, especially in regions with stigma or limited access to care. Without intervention, many students experienced chronic or worsening symptoms.

This trend reflects increasing intolerance to negative emotions, ambiguity, and delayed gratification.^38-40^ Emotional distress often stems from a perception that effort outweighs reward, influencing how young adults interpret work demands.^41^ Reward-based parenting has shaped a generation accustomed to emotional ease and protective enablement and flexibility.^42,43^ Such characteristics reduce resilience and perseverance.^2^

Modern protective-enabling parenting styles impact intrinsic motivation^17^; particularly perceptions of autonomy, competence, and relatedness, as outlined in Self-Determination Theory.^44^ When such needs are disrupted or unmet, deficits in this triad of needs predict depression, low self-efficacy, risky behaviors, and interpersonal difficulties.^45^ These challenges potentially affect care-giving professions, where emotional demands and independence remain high.^38^ Failure to develop core motivational traits can lead to burnout and compassion fatigue, diminishing empathy and performance.^46^ Thus, modern parenting styles have contributed to emotional vulnerabilities in emerging adults by impairing autonomy, competence, and relatedness, which are core needs in Self-Determination Theory.^17,40^

Compounding this issue is technology’s omnipresence, which has shaped effortless lifestyles, cognitive shortcuts, and instant gratification.^38,47,48^ Cognitive, physical, and social offloading from excessive screen use predicts depressive symptoms, often driven by loneliness and social media overuse.^31,49,50^ Social avoidance behaviors and reduced tolerance for effortful interactions reflect deeper emotional vulnerability among young adults.^17,50^ Unhealthy lifestyle habits characterized by sedentary behavior, poor diet, tobacco and substance use, and energy drink overuse raise such mental and physical health risks.^25,51,52^

Evidence shows that disconnecting from mobile internet can improve psychological well-being. Castelo et al^53^ conducted a randomized trial where participants had mobile internet blocked for 2 weeks, creating a “flip-phone” environment while still allowing calls and texts. Desktop internet remained available. This intervention led to better mental health, well-being, cognitive performance, and life quality—driven by more time outdoors, socializing, and exercising. Notably, depression symptoms dropped by 56%, surpassing typical antidepressant effects and mirroring gains seen with cognitive behavioral therapy.

Understanding the influences of upbringing and the perceived value of pastimes on today’s recruits helps explain retention challenges, especially in health settings. Institutions face ongoing barriers to professional integration, including staff shortages, rapid human resource management, fast-paced care delivery, and pressure to promote patient self-care.^12^ These strains can lead to patient dissatisfaction.^54,55^

## Mental Health Literacy in Education and Health Sectors

Mental health literacy refers to knowing how to maintain well-being, recognize disorders, seek help, and reduce stigma.^56-58^ Although mental health literacy informs both self-care and the needs of others,^58^ it does not translate into wellness without personal effort and environmental supports. Wellness promises team cohesion and functioning in care settings.^59^ Psychiatry, as a discipline, now promotes lifestyle medicine as a pathway to learned wellness.^25^ The lack of methodological homogeneity makes it hard to establish evidence-based conclusions about the efficacy of MHL programs. The task of emerging adulthood is to develop an identity by establishing meaning in life, which facilitates resilience.^17^ Learned wellness represents an effortful step beyond mental health literacy.^58^ It thus requires environmental supports toward autonomy (control), competence (skills), and relatedness (having an “other” orientation) toward establishing a purpose in life, larger than oneself.^40,44^

Education and health sectors must integrate these needs and values into workforce policies to match recruits with institutional missions.^12,19^ Helping professions support vulnerable populations dealing with trauma, abuse, and oppression, requiring resilience often lacking in newer generations.^60^ Junior staff raised with protective parenting and tech-heavy lifestyles may find training demanding compared with older peers.^61^

## Workforce Vulnerabilities and Integration Challenges

When the 3 core psychological needs of autonomy, competence, and relatedness are not fostered, particularly in high-stress environments which characterize health—individuals experience increased secondary stress.^62^ New-generation health recruits, particularly frontline workers, face significant vulnerabilities related to professional integration.^63-65^ One of the primary risks in their work environment is secondary traumatic stress, which arises from the emotional and psychological toll of vicarious exposure to trauma and suffering.^66^ Prolonged and intense exposure to others’ distress can severely compromise both mental and physical health, often leading to post-traumatic stress injury symptoms or disorder.^64^

Secondary traumatic stress frequently contributes to burnout syndrome, a widespread occupational hazard in the health care sector, especially among nurses and physicians.^63^ Burnout is characterized by emotional exhaustion, mental fatigue, and depersonalization or cynicism, accompanied by negative perceptions of personal accomplishment, workplace environment, and colleagues.^67^ These symptoms impair personal and occupational functioning, ultimately affecting work quality, retention, and staff turnover.^68^ Secondary trauma risks affect attrition, or wanting to leave the career or workplace. The most reliable predictors of worker vulnerability are perceptions of work-place stress and fatigue deriving from perceptions of lack of support.^64,66^ Reduced staffing exacerbates perceived workloads, creating a vicious cycle that further depletes human resources.^69^

Decades of research with prior generations have underscored serious mental health concerns in the health sector, with increasing rates of burnout.^70^ Chirico et al^63^ conducted an umbrella review of systematic reviews and meta-analyses of the existing literature until 2020, thus unconfounded by the pandemic. Among the 43 studies that met the full inclusion criteria, a higher prevalence of burnout syndrome was highest among nurses, younger health care workers, and trainees. Prolonged night shifts, length of experience, and exposure to traumatic events figured prominently as organizational risk factors. The high-pressure nature of frontline work, compounded by understaffed environments and the risk of professional errors, further amplifies this risk.^68^ Negative environmental perceptions, where distress becomes psychological strain, charts a developmental course toward professional disintegration.^64^

Underlying both burnout and secondary trauma is compassion fatigue, defined as emotional and physical exhaustion resulting from chronic exposure to suffering. This condition diminishes empathy and undermines professional effectiveness, leading to increased treatment errors and declining care quality.^71^ Compassion fatigue also influences caregiver emotional states, beliefs, and behaviors, straining the caregiving workforce.^72,73^ The emotional distress associated with such challenges has been linked to significantly higher suicide rates among health professionals, including physicians, veterinarians, pharmacists, and nurses, even after accounting for sociodemographic factors.^56,74,75^

Being a hardy clinical worker requires intrinsic motivation, and the cumulative impact of prolonged, intense exposure to the distress of others can undermine perceptions of autonomy (feeling little control), competence (feeling low empathy skills), and relatedness (feeling low support from others).^44^ Accordingly, perceptions of flexibility and control, mentorship and growth experiences, and strong workplace connections and a sense of belonging would be crucial for resilience.^76^

In workplace settings, particularly in health care, strain is exacerbated by secondary trauma and burnout, which amplify emotional exhaustion and disrupt interpersonal dynamics.^12^ Perceptions of low support and heavy workloads, particularly for younger generations entering the workforce, further contribute to negative perceptions of accomplishment and increased emotional distress.^63^ Emotional exhaustion strains relationships with coworkers and patients and disrupts workplace dynamics. Such challenges not only diminish the quality of care but also lead to broader consequences for health institutions beyond the individual impact on personal and family relationships.^68^

### Stress and Strain: Broader Mechanisms Underpinning Workplace Challenges

Burnout and compassion fatigue exemplify specific occupational stress responses, but these are part of a broader spectrum of stress and strain that affect individuals across various domains. Stress refers to the body’s response to challenges perceived as exceeding adaptive resources, manifesting as physical, emotional, or psychological reactions such as increased heart rate, anxiety, or irritability.^76^ It involves perceptions and attributions regarding external stressors, such as work pressures, personal difficulties, or significant life changes. It can cause increased heart rate, muscle tension, and emotional responses related to anxiety or irritability. On the other hand, strain is the psychological wear and tear resulting from prolonged or intense exposure to stressors, leading to emotional and cognitive consequences when coping mechanisms are insufficiently.^77^ Psychological stress and its consequence, strain, is preventable and rooted in mental health literacy.^69^

Fundamental theories of motivation speak directly to psychological growth, social integrity, and well-being.^44,78^ Workplace motivation is vital for providing high levels of satisfaction among trainees and employees who seek ways to enhance care of the vulnerable under their care. Although younger age is generally considered the most powerful predictor of vulnerability to compassion fatigue in professions, it remains confounded with field experience and social relationships at work.^63^ Experience and relationships take time, as does perceived purpose in life, which represents a powerful protective factor against burnout and secondary traumatic stress.^79^

### Addressing Institutional Gaps

Hospitals operate under the assumption that clinicians have been trained by educational institutions to provide optimal care and guidance in relational contexts such as counseling, operationalizing basic needs and support, and mediating/advocating for individuals under their care.^12^ Clinical training activities assume that new recruits have the requisite skills for professional integration.^19^ Both hospitals and training institutions typically omit self-care and well-being training for caregivers even though such professionals experience situations that create vulnerability daily.^68^ This is a pressing concern for younger adults in the workforce, whose values and aspirations increasingly drive them to call on employers to prioritize preventive emotional health at an institutional level.^20^

To address challenges related to stress responses and institutional gaps, institutions should implement proactive strategies that not only support professional integration but also equip workers with the psychological resilience needed to thrive in challenging environments.^80^ The aim would be to foster resilience, manage workplace stressors effectively, and reduce the risk of strain for all workers, and especially incoming recruits. This cannot be achieved with self-determination theory alone, but rather an approach that considers and interventions that are grounded in subjective experiences of well-being.

### Resilience-Building Strategies

Agency toward well-being involves intentional thoughts and actions that support personal fulfillment.^80^ Subjective well-being can be approached as (1) being happy *with* life or (2) being happy *in* life.^30,81^ The former reflects long-term satisfaction (eudaimonia); the latter emphasizes momentary pleasure (hedonia). Both perspectives stem from Self-Determination Theory, which sees resilience as a motivational force that gives life meaning.^44,80^

Soren and Ryff^30^ offer a 6-factor model for sustained well-being: self-acceptance, positive relationships, autonomy, environmental mastery, purpose, and personal growth—key for developing resilience, especially in career ambivalence.^78^ Seligman’s model prioritizes 5 components of flourishing: emotions, engagement, relationships, meaning, and accomplishment, focusing on “values-in-action” and personal strengths for thriving.^81^

These frameworks suggest well-being is teachable and adaptable,^80^ with both short- and long-term focus reducing compassion fatigue.^82,83^ Institutions must support resilience and self-compassion through environment design,^84,85^ and promote protective activities related to empathy, gratitude, emotion regulation, and mental flexibility to strengthen teamwork and clinician performance.^61^ Cultivating growth-centered mindsets enhances mental health by turning difficulty into opportunity.^86^ Supporting self-efficacy in trainees and staff could address negative workplace perceptions and mitigate attrition.^87,88^

Work and school environments in health can also leverage strengths, passions, and talents that can potentially improve tolerance to negative emotion.^25,78,80^ Self-awareness of habitual predispositions has potential in facilitating person-environment fit for both individuals and institutions, thus fostering more effective collaboration and workplace communication.^89^ Complementing awareness of character strengths (measurable at Authentic Happiness | Authentic Happiness) are personality profiles such as Five-Factor Model or Myers-Briggs Type Indicator to optimize workplace integration.^90^ Knowledge of character strengths facilitates identifying where individuals find themselves strong and, in turn, enhances positive and constructive approaches to finding solutions. Knowledge of one’s personality profile assists the person-environment fit in environments that seek constructive solutions. Learning about one’s character traits, strengths, and personality profile also generates insight on which kinds of leadership to expect and leverage from individuals and teams.^91^ These affect, and are affected by lifestyle and work habits.^17^ Although valid and reliable for clinical purposes with person-centered interventions, these measures might be limited by limited predictive power regarding individual clinical outcomes, such as treatment adherence or relapse risk.^89,90^

## Clinical Principles in Action

Twelve billion working days are lost to depression and anxiety alone, not only costing the global economy 1 trillion annually but also reducing chances of human flourishment and productivity.^65^ Psychology centers in universities and human resource departments in health institutions are preoccupied with the increasing prevalence, of which, for many, become lifelong struggles beyond graduation or the work orientation period in a new career.^22^

Employers of university-educated health workers must not expect such mental health risks to disappear upon graduation.^56,92^ The next challenge in young adulthood is professional integration.^19,56^ Past or existing history of mental disorder predicts risk of unsuccessful integration when faced with the obligations of service in health care settings.^56^

### Affective/Anxiety Disorders

In more recent young generations, affective and anxiety disorders have a populational prevalence of one-fifth and one-sixth of the adult population, respectively.^24^ Caregiving staff with a history of resolved or unresolved affective and anxiety disorders may exhibit specific characteristics that influence their emotional and information processing related to emotional instability and negative perceptions of ambiguity in the environment, respectively. This affects interactions with institutions, colleagues, and patients.^93^

The most challenging and common features between both disorders are ruminative thinking, concentration difficulties, negativity bias, and dramatizing.^76,93,94^ Consequently, individuals affected are at risk of being more sensitive to criticism, patient outcomes, or feedback from colleagues and superiors. They also have a propensity for having trouble managing emotions, and vulnerability to mood swings and emotional reactivity in energy-demanding situations. Looming or residual negative emotions (sadness, disinterest, fear, apprehension, anger) might contribute to feelings of inadequacy, self-doubt, or imposter syndrome (fear of underperformance or decision paralysis). Finally, performance anxiety, associated with both affective and anxiety disorders, might be reflected by intense worry about making mistakes or failing to meet professional expectations.

Personal battles with self-confidence could hinder effective collaboration with colleagues. Behaviorally, underlying insecurity may generate an excessive compensatory drive to meet institutional standards, leading to overworking or underworking to generate confidence or distract from inner struggles with pessimism, respectively. Conversely, they might avoid/delay tasks or situations perceived as emotionally challenging, which complexifies decision-making and conflict resolution in critical situations. There might be a tendency to withdraw from colleagues or angrily engage when frustrated or under pressure. This generates challenges in teamwork and empathetic patient care.

Two chief strengths in this risk group are worthy of note.^93^ Their intense awareness of emotional content, often rooted in their life course experiences, renders persons with a history of affective or anxiety disorders potentially empathetic caregivers. Their intense reliance on feedback from supervisors, colleagues, or patients to affirm their self-worth can be leveraged to improve their emotional experiences and encourage a growth perspective.^78^

Character traits can be leveraged to help manage and maximize functioning in work settings. Developing a character strengths-based mindset predicts lower rates of anxiety and depression in individuals with a history of affective and anxiety disorders.^81^ Enhanced awareness of character strengths and personality profiles can be strategically used to enhance short and long-term subjective well-being.^95^ Knowledge of one’s unique strengths can be critical for enhancing social relationships and quality of life. In fact, Williams and Kumar^96^ recently found that the effectiveness of such interventions are mediated by increased global self-worth.

### Neurodevelopmental Disorders and Institutional Fit

Typically emerging in early childhood, neurodevelopmental conditions often accompany distinctive individual strengths that can enrich workplace environments when properly supported. These strengths may include heightened sensitivity to detail, deep focus, creativity, and resilience traits that, when aligned with appropriate roles and settings, contribute meaningfully to team dynamics and innovation.^95^ Having had a childhood neurodevelopmental disorder might mean being more reactive to trauma and experiencing intense difficulties in sensory processing, such as sound, light, and spatial input.^97^ Individuals with such disorders are more likely to have particular difficulties with motor skills, making them more physically awkward than unaffected people.^98^ There are also higher risks of comorbid symptoms or disorders of a depressive and anxious nature.^95^ Such characteristics can create challenges in a workplace setting characterized by ever-changing social interactions, complex instructions, coordinated movements, protocol interruptions and modifications, and time pressure. Nevertheless, enhanced awareness of strengths of character and personality can be strategically leveraged to enhance short- and long-term subjective well-being at home and at work.^98^

Among neurodevelopmental conditions, ADHD is one of the most prevalent, affecting approximately 7% of the global population.^99^ Many individuals with ADHD exhibit strengths such as curiosity, zest, and creativity. These represent traits that can be powerful assets in dynamic and high-pressure environments.^95^ Because they need to feel high levels of arousal to achieve a state of “flow,” many feel positive emotion in high-stakes settings such as the emergency room or first responding. This is a hidden strength that often goes unnoticed by individuals and workplaces.^100^ ADHD has also been related to creative thinking, resistance to cultural or peer pressure, and improvisation when faced with obstacles.^99^ The main symptoms are related to executive function deficit, with or without hyperactive or impulsive behavior. In activities of daily living, this could translate into difficulties in setting realistic goals, organizing tasks, and maintaining focus in the presence of disturbances.^101^

Transitioning to another major neurodevelopmental condition, Autism Spectrum Disorder (ASD) affects approximately 2% to 3% of the population.^102^ Individuals on the autism spectrum often develop intense expertise in specific fields, driven by strong passion and exceptional memory skills.^103^ The character strengths most frequently reported include honesty, appreciation of beauty and excellence, love of learning, fairness, and kindness.^95^ In fact, higher levels of life satisfaction have been associated with the strengths of gratitude, hope, and honesty. Research has also highlighted creativity, concrete (as opposed to abstract) perspective-taking, and a focus on details as potential strengths.^104^ The main features of this spectrum include, but are not limited to, an unusual or unique way of communicating.^101^ They can be less expressive in terms of facial expressions and might have difficulty interpreting facial expressions in other people, and have restricted interests and behaviors.^102^ This means that such individuals may have difficulty partaking in conversations and activities they do not perceive as interesting, which can generate misunderstandings in communication. Affected individuals typically achieve a state of flow and feel stimulated in work settings with predictability and routines. This means that they fit well in contexts and objectives characterized by activities with recurring protocols and patient needs. Such posts that are often difficult to fill when employers do not think about person-environment fit strategies as an initial human resource strategy.^89^

Closely related to ADHD and ASD, learning disabilities affect nearly one-sixth of the global population.^105^ Almost one-sixth of the global population has a past or recent history of learning disability.^106^ In people with a current or previous history of learning disability, secondary strengths such as persistence, grit, empathy, social intelligence, and creativity often emerge through coping with past challenges with learning. Interventions that enhance strengths related to hope and self-regulation have been associated with better coping mechanisms in such individuals.^105^ Having developed unique learning-related strategies in childhood and adolescence tends to foster exceptional talents or abilities that can facilitate work skills in this group, such as thinking “outside the box” and perceiving the “emotional context” in a situation. This can, if encouraged, facilitate the identification of the needs of others.^107^ This disorder affects the ability to process, store, and communicate information. Because they share a genetic etiology,^106^ people affected often experience symptoms that might be comorbid with other neurodevelopmental disorders. As a result, they can be difficult to distinguish from individuals with ADHD and Autism. Many people with an existing neurodevelopmental disorder achieve the diagnostic criteria for a comorbid learning disability.^105^

## Implications for Policy and Practice

Indeed, there are challenges that Gen Z faces in the workplace, such as intolerance to negative emotion, feelings of alienation or exclusion associated with neurodevelopmental disorders, burnout and secondary trauma risks, and difficulty navigating traditional hierarchies. Each recruit, whether a trainee or a professional, is a valuable resource deserving respect for their unique strengths, talents, and interests.^108^ Worker well-being should remain central to policies aimed at fostering a person-environment fit in early adulthood.^109^ Unlike previous generations, millennials and Gen Z thrive in individual-centered or employee-focused environments. Consequently, a realignment of institutional priorities toward promoting individual development—alongside an essential focus on work-life considerations such as social relationships, physical activity, and child-care—is needed. These elements are key motivational factors for sustainable growth and the retention of incoming professionals.^15^

To operationalize these priorities, organizations can adopt trauma-informed onboarding models that emphasize psychological safety, transparency, and trust-building from day 1. Frameworks such as the CSA Z1003 Standard for Psychological Health and Safety in the Workplace or SAMHSA’s trauma-informed care principles offer structured approaches to cultivating environments where Gen Z employees feel seen, supported, and empowered (eg, CSA Z1003 and SAMHSA projects).^109,110^ By adapting and shifting toward the needs of this new generation of recruits, employers and training institutions can unlock their potential. Rather than expecting them to conform to outdated structures, this approach enriches their mental health and cultivates positive emotions and feelings of accomplishment among health workers.

Preventive programs that help workers understand their unique personality profiles, value their character strengths, teach stress management, and cultivate the relationship between effort and growth as a reward—rather than solely focusing on results—represent a valuable and long-lasting investment.^85,108^ Fostering and maintaining optimal bio-psycho-social wellness through evidence-based frameworks provides the foundation for sustainable resilience against risks such as compassion fatigue.^60^

## Conclusions

This commentary aimed to provide an integrative theoretical and clinical position that synthesizes key findings from clinical research into a well-structured framework, informing practice, policy, and future research. At its core, the argument centers on the principle of person-environment fit—the alignment between individual traits, values, and needs with the demands and culture of the workplace.^89^ Substantial numbers of health care trainees and new recruits face a developmental intolerance to negative emotions stemming from the responsibility challenges of transitioning to adulthood. To address this, institutions must prioritize work-life balance and foster an atmosphere that values growth, incremental performance, and psychological safety—key conditions for achieving optimal fit between individuals and their work environments.^18^ Health institutions must integrate well-being strategies into their policies to combat the workforce in crisis.^60^ This necessitates emphasizing mental wellness through targeted training, fostering resilience, and promoting work-life balance—not as isolated interventions, but as part of a broader strategy to enhance person-environment fit and retain young professionals.^89^ Furthermore, institutions of higher education and employers must shift their focus beyond performance metrics to actively support wellness from the outset, cultivating inclusive environments that recognize and respond to diverse student and workforce needs. A growth perspective must be cultivated, promoting increased retention and long-term engagement.^111^

New recruits require a clear understanding of their individual values, talents, and passions to thrive effectively in academic and professional settings.^17,108^ Preventive universal support for mental health literacy and targeted intervention strategies toward wellness are crucial—but their effectiveness hinges on how well they help individuals find roles and environments that reflect their strengths and aspirations. Finally, cultivating a work environment that values personal relationships within the work-life equation enhances both personal and social fulfillment.^111^ Workplaces that foster supportive vertical and horizontal relationships create conditions for thriving, as hallmarks of strong person-environment fit, and are associated with significantly higher retention rates.^60^

Taken together, this approach—grounded in the principle of person-environment fit—offers a cohesive framework for mitigating attrition risk, improving educational outcomes, and enhancing psychosocial functioning within the young adult workforce.^89^ It invites institutions to move beyond reactive measures and toward proactive, strengths-based strategies that align people with environments where they can flourish. To truly address the challenges that Generation Z faces which affect the workforce, institutional leaders must move beyond mental health literacy rhetoric and concretely foster individual and team wellness support in structural and policy reforms.

## References

[1] Kim J Chae D Yoo JY . Reasons behind Generation Z nursing students’ intentions to leave their profession: a cross-sectional study. INQUIRY. 2021;58:1-8. doi:10.1177/0046958021999928 PMC7940812 33660536

[2] Zhang W Ma X Xiao Q Yu S Zhang M Wang X . Career development and occupational disease in Chinese nurses: a cross-sectional study. INQUIRY. 2022;59:00469580221092819. doi:10.1177/00469580221092819 PMC9016528 35416729

[3] Grant MJ Booth A . A typology of reviews: an analysis of 14 review types and associated methodologies. Health Inf Libr J. 2009;26(2):91-108. doi:10.1111/j.1471-1842.2009.00848.x 19490148

[4] Thapar A Pine DS Leckman JF Scott S Snowling MJ Taylor E , eds. Rutter’s Child and Adolescent Psychiatry. 7th ed. Wiley-Blackwell; 2022.

[5] Cucina JM Byle KA Martin NR Peyton ST Gast IF . Generational differences in workplace attitudes and job satisfaction: lack of sizable differences across cohorts. J Manag Psychol. 2018;33(3):246-264. doi:10.1108/JMP-03-2017-0115

[6] McGorry PD Mei C Dalal N , et al The Lancet Psychiatry Commission on youth mental health. Lancet Psychiatry. 2024;11(9):731-774. doi:10.1016/S2215-0366(24)00163-9 39147461

[7] Huysentruyt M de los Ángeles Gutiérrez M Algan Y . Bridging Social Capital and Trust: A Research Agenda. HEC Paris; 2025. Accessed February 3, 2025. https://www.hec.edu/sites/default/files/documents/Social-Capital-and-Trust-HEC-S%26O-Institute-2025_compressed.pdf

[8] Heckman JJ Kautz T . Hard evidence on soft skills. Labour Econ. 2012;19(4):451-464. doi:10.1016/j.labeco.2012.05.014 23559694 PMC3612993

[9] Ng R Indran N . Societal perceptions of caregivers linked to culture across 20 countries: evidence from a 10-billion-word database. PLoS One. 2021;16(7):e0251161. doi:10.1371/journal.pone.0251161 PMC8248619 34197470

[10] Parent-Lamarche A Hallée Y . Exploring the effects of predominantly female jobs on demands and resources at work and consequently on health and performance in the Province of Québec, Canada. Int Arch Occup Environ Health. 2023;96(9):1267-1281. doi:10.1007/s00420-023-02005-3 37599309

[11] Deming DJ . Four facts about human capital. J Econ Perspect. 2022;36(3):75-102. doi:10.1257/jep.36.3.75

[12] Minzlaff KA Palmer S Fillery-Travis A . The significance and challenges of turnover and retention of millennial professionals. J Work-Appl Manag. 2025;17:34-49. doi:10.1108/jwam-07-2023-0062.

[13] Fry R . Millennials outnumbered Boomers in 2019. Pew Research Center. Published April 28, 2020. Accessed February 3, 2025. https://www.pewresearch.org/short-reads/2020/04/28/millennials-overtake-baby-boomers-as-americas-largest-generation/

[14] Scandurra R Cefalo R Kazepov Y . Drivers of youth labour market integration across European regions. Soc Indic Res. 2021;154(3):835-856. doi:10.1007/s11205-020-02549-8

[15] Halder SR Mukherjee A Sahay K Rajak MP Mohan A Sagar S . Decoding gen Z: unraveling workforce preferences, consumer behavior, and financial decision-making in the IR 4.0. Econ Sci. 2025;21(1):258-268. doi:10.69889/dmv67406

[16] Suhara A Sabardini SE Darma M Sayuti AM Swastika MT . Conflict management strategies between generations in the workplace: perspectives from millennials and baby boomers. J Acad Sci. 2025;2(2):513-521. doi:10.59613/jvggv153

[17] Twenge JM . Generations: The Real Differences Between Gen Z, Millennials, Gen X, Boomers, and Silents—and What They Mean for America’s Future. Simon & Schuster; 2023.

[18] Trifan VA Pantea MF . Shifting priorities and expectations in the new world of work: insights from millennials and generation Z. J Bus Econ Manag. 2024;25(5):1075-1096. doi:10.3846/jbem.2024.22469

[19] Ocean N Meyer C . Satisfaction and attrition in the UK healthcare sector over the past decade. PLoS One. 2023;18(4):e0284516. doi:10.1371/journal.pone.0284516 PMC10101409 37053234

[20] Deloitte. The Deloitte Global 2022 Gen Z and millennial survey: top concerns among Gen Zs and millennials. Published 2022. Accessed February 3, 2025. https://www2.deloitte.com/global/en/pages/about-deloitte/articles/genzmillennialsurvey.html

[21] Kakemam E Liang Z Janati A Arab-Zozani M Mohaghegh B Gholizadeh M . Leadership and management competencies for hospital managers: a systematic review and best-fit framework synthesis. J Healthc Leadersh. 2020;12:59-68. doi:10.2147/JHL.S265825 32801985 PMC7383104

[22] Mason A Rapsey C Sampson N , et al Prevalence, age of onset, and course of mental disorders among 72,288 first-year university students from 18 countries in the World Mental Health International College Student (WMH-ICS) initiative. J Psychiatr Res. 2025;183:225-236. doi:10.1016/j.jpsychires.2025.02.016 40010072 PMC11926851

[23] Solmi M De Toffol M Kim JY , et al Balancing risks and benefits of cannabis use: umbrella review of meta-analyses of randomised controlled trials and observational studies. BMJ. 2023;382:e072348. doi:10.1136/bmj-2022-072348 PMC10466434 37648266

[24] Shorey S Ng ED Wong CHJ . Global prevalence of depression and elevated depressive symptoms among adolescents: a systematic review and meta-analysis. Br J Clin Psychol. 2022;61(2):287-305. doi:10.1111/bjc.12333 34569066

[25] Firth J Solmi M Wootton RE , et al A meta-review of “lifestyle psychiatry”: the role of exercise, smoking, diet, and sleep in the prevention and treatment of mental disorders. World Psychiatry. 2020;19(3):360-380. doi:10.1002/wps.20773 32931092 PMC7491615

[26] Barkley RA Dawson G . Higher risk of mortality for individuals diagnosed with autism spectrum disorder or attention-deficit/hyperactivity disorder demands a public health prevention strategy. JAMA Pediatr. 2022;176(4):e216398. doi:10.1001/jamapediatrics.2021.6398 PMC10072169 35157011

[27] Cerolini S Zagaria A Franchini C , et al Psychological counseling among university students worldwide: a systematic review. Eur J Investig Health Psychol Educ. 2023;13(9):1831-1849. doi:10.3390/ejihpe13090133 PMC10528000 37754472

[28] Bushnell GA Rynn MA Gerhard T , et al Drug overdose risk with benzodiazepine treatment in young adults: comparative analysis in privately and publicly insured individuals. Addiction. 2024;119(2):356-368. doi:10.1111/add.16359 37816665 PMC10838605

[29] Cuijpers P Miguel C Harrer M , et al Cognitive behavior therapy vs. Control conditions, other psychotherapies, pharmacotherapies and combined treatment for depression: a comprehensive meta-analysis including 409 trials with 52,702 patients. World Psychiatry. 2023;22(1):105-115. doi:10.1002/wps.21069 36640411 PMC9840507

[30] Soren A Ryff CD . Meaningful work, well-being, and health: enacting a eudaimonic vision. Int J Environ Res Public Health. 2023;20(16):6570. doi:10.3390/ijerph20166570 37623156 PMC10454804

[31] Collins S Hoare E Allender S , et al A longitudinal study of lifestyle behaviours in emerging adulthood and risk for symptoms of depression, anxiety, and stress. J Affect Disord. 2023;327:244-253. doi:10.1016/j.jad.2023.02.010 36754097

[bibr32-00469580251399302] Duffy A Keown-Stoneman C Goodday S , et al Predictors of mental health and academic outcomes in first-year university students: identifying prevention and early-intervention targets. BJPsych Open. 2020;6:e46. doi:10.1192/bjo.2020.24 PMC7331085 32381150

[bibr33-00469580251399302] Oswalt SB Lederer AM Chestnut-Steich K Day C Halbritter A Ortiz D . Trends in college students’ mental health diagnoses and utilization of services, 2009-2015. J Am Coll Health. 2020;68(1):41-51. doi:10.1080/07448481.2018.1515748 30355071

[bibr34-00469580251399302] Pringsheim T Stewart DG Chan P Tehrani A Patten SB . The pharmacoepidemiology of psychotropic medication use in Canadian children from 2012 to 2016. J Child Adolesc Psychopharmacol. 2019;29(10):740-745. doi:10.1089/cap.2019.0018 31355670

[35] Wiens K Bhattarai A Pedram P , et al A growing need for youth mental health services in Canada: examining trends in youth mental health from 2011 to 2018. Epidemiol Psychiatr Sci. 2020;29:e115. doi:10.1017/S2045796020000281 PMC7214527 32299531

[36] Eskin M Sun JM Abuidhail J , et al Suicidal behavior and psychological distress in university students: a 12-nation study. Arch Suicide Res. 2016;20(3):369-388. doi:10.1080/13811118.2015.1054055 26954847

[37] Mortier P Cuijpers P Kiekens G , et al The prevalence of suicidal thoughts and behaviours among college students: a meta-analysis. Psychol Med. 2018;48(4):554-565. doi:10.1017/S0033291717002215 28805169

[38] Monaco AP . An epigenetic, transgenerational model of increased mental health disorders in children, adolescents, and young adults. Eur J Hum Genet. 2021;29(3):387-395. doi:10.1038/s41431-020-00726-4 32948849 PMC7940651

[bibr39-00469580251399302] Sahib A Chen J Cárdenas D Calear AL . Intolerance of uncertainty and emotion regulation: a meta-analytic and systematic review. Clin Psychol Rev. 2023;101:102270. doi:10.1016/j.cpr.2023.102270 36965452

[40] Silvers JA Peris TS . Research review: the neuroscience of emerging adulthood - reward, ambiguity, and social support as building blocks of mental health. J Child Psychol Psychiatry. 2023;64(7):989-997. doi:10.1111/jcpp.13776 36878602

[41] Porru F Robroek SJW Bültmann U Portoghese I Campagna M Burdorf A . Mental health among university students: the associations of effort-reward imbalance and overcommitment with psychological distress. J Affect Disord. 2021;282:953-961. doi:10.1016/j.jad.2020.12.183 33601740

[42] McCoy SS Dimler LM Rodrigues L . Parenting in overdrive: a meta-analysis of helicopter parenting across multiple indices of emerging adult functioning. J Adult Dev. 2025;32:222-245. doi:10.1007/s10804-024-09496-5

[43] Hwang W Jung E Fu X , et al Is helicopter parenting related to college students’ mental health? A typological and cross-cultural approach. Fam Relat. 2023;72(4):2215-2233. doi:10.1111/fare.12802

[44] Ryan RM Ryan WS Di Domenico SI Deci EL . The nature and the conditions of human autonomy and flourishing: self-determination theory and basic psychological needs. In: Ryan RM , ed. The Oxford Handbook of Human Motivation. 2nd ed. Oxford University Press; 2019;89-110.

[45] Gomes SB Deuling JK . Family influence mediates the relation between helicopter-parenting and millennial work attitudes. J Manag Psychol. 2019;34(1):2-17. doi:10.1108/JMP-12-2017-0450

[46] LeBlanc JE Lyons ST . Helicopter parenting during emerging adulthood: consequences for career identity and adaptability. Front Psychol. 2022;13:886979. doi:10.3389/fpsyg.2022.886979 36211870 PMC9532949

[47] Sanders T Noetel M Parker P , et al An umbrella review of the benefits and risks associated with youths’ interactions with electronic screens. Nat Hum Behav. 2024;8(1):82-99. doi:10.1038/s41562-023-01712-8 37957284

[48] Krokstad S Weiss DA Krokstad MA , et al Divergent decennial trends in mental health according to age reveal poorer mental health for young people: repeated cross-sectional population-based surveys from the HUNT study, Norway. BMJ Open. 2022;12(5):e057654. doi:10.1136/bmjopen-2021-057654 PMC9119156 35584877

[49] Elhai JD Gallinari EF Rozgonjuk D Yang H . Depression, anxiety, and fear of missing out as correlates of social, non-social, and problematic smartphone use. Addict Behav. 2020;105:106335. doi:10.1016/j.addbeh.2020.106335 32062337

[50] Marttila E Koivula A Räsänen P . Does excessive social media use decrease subjective well-being? A longitudinal analysis of the relationship between problematic use, loneliness, and life satisfaction. Telematics Inform. 2021;59:101556. doi:10.1016/j.tele.2020.101556

[51] Sampogna G Lovisi GM Zinno F , et al Mental health disturbances and related problems in Italian university medical students from 2000 to 2020: an integrative review of qualitative and quantitative studies. Medicinar. 2020;57(1):11. doi:10.3390/medicina57010011 PMC7823352 33374475

[52] Welsh JW Shentu Y Sarvey DB . Substance use among college students. Focus. 2019;17(2):117-127. doi:10.1176/appi.focus.20180037 31975967 PMC6527004

[53] Castelo N Kushlev K Ward AF Esterman M Reiner PB . Blocking mobile internet on smartphones improves sustained attention, mental health, and subjective well-being. PNAS Nexus. 2025;4(2):af017. doi:10.1093/pnasnexus/pgaf017 PMC11834938 39967678

[54] Bridgeman PJ Bridgeman MB Barone J . Burnout syndrome among health care professionals. Am J Health Syst Pharm. 2018;75(3):147-152. doi:10.2146/ajhp170460 29183877

[55] Zambrano-Chumo L Guevara R . Psychological capital and turnover intention: the mediating role of burnout among healthcare professionals. Int J Environ Res Public Health. 2024;21(2):185. doi:10.3390/ijerph21020185 38397676 PMC10888532

[56] Ghio L Patti S Piccinini G , et al Anxiety, depression, and risk of post-traumatic stress disorder in health workers: the relationship with burnout during COVID-19 pandemic in Italy. Int J Environ Res Public Health. 2021;18(18):9929. doi:10.3390/ijerph18189929 34574851 PMC8469269

[bibr57-00469580251399302] Meredith LS Bouskill K Chang J Larkin J Motala A Hempel S . Predictors of burnout among US healthcare providers: a systematic review. BMJ Open. 2022;12(8):e054243. doi:10.1136/bmjopen-2021-054243 PMC9422884 36008065

[58] Mansfield R Patalay P Humphrey N . A systematic literature review of existing conceptualisation and measurement of mental health literacy in adolescent research: current challenges and inconsistencies. BMC Public Health. 2020;20:607. doi:10.1186/s12889-020-08734-1 32357881 PMC7195735

[59] McGuier EA Kolko DJ Aarons GA , et al Teamwork and implementation of innovations in healthcare and human service settings: a systematic review. Implement Sci. 2024;19:49. doi:10.1186/s13012-024-01381-9 39010100 PMC11247800

[60] Vîrgă D Baciu EL Lazăr TA Lupșa D . Psychological capital protects social workers from burnout and secondary traumatic stress. Sustainability. 2020;12(6):2246. doi:10.3390/su12062246

[61] Perry MA Creavey K Arthur E Chance Humer J Lundgren PJ Rivera I . Cultivating emotional intelligence in child welfare professionals: a systematic scoping review. Child Abuse Negl. 2020;110:104438. doi:10.1016/j.chiabu.2020.104438 32164944

[62] Ravn I . The existential challenges of stress, trauma, and psychopathology and their integration to the self: a self-determination theory perspective. Trends Psychol. 2024;32:1-15. doi:10.1007/s43076-024-00373-4

[63] Chirico F Afolabi AA Ilesanmi OS , et al Prevalence, risk factors, and prevention of burnout syndrome among health care workers before the COVID-19 pandemic: an umbrella review of systematic reviews and meta-analyses. J Health Soc Sci. 2021;6(4):465-491. doi:10.19204/2021/prvl3

[bibr64-00469580251399302] Hall LH Johnson J Watt I Tsipa A O'Connor DB . Healthcare staff wellbeing, burnout, and patient safety: a systematic review. PLoS One. 2016;11(7):e0159015. doi:10.1371/journal.pone.0159015 PMC4938539 27391946

[65] World Health Organization. Burn-out an “occupational phenomenon”: International Classification of Diseases. Published 2024. Accessed February 3, 2025. https://www.who.int/mental_health/evidence/burn-out/en/

[66] Wolotira EA . Trauma, compassion fatigue, and burnout in nurses: the nurse leader’s response. Nurse Lead. 2023;21(2):202-206. doi:10.1016/j.mnl.2022.04.009 35582625 PMC9098943

[67] Maslach C Jackson SE . The measurement of experienced burnout. J Occup Behav. 1981;2(2):99-113. doi:10.1002/job.4030020205

[68] Kelly L . Burnout, compassion fatigue, and secondary trauma in nurses: recognizing the occupational phenomenon and personal consequences of caregiving. Crit Care Nurs Q. 2020;43(1):73-80. doi:10.1097/CNQ.0000000000000293 31789880

[69] Helfrich CD Simonetti JA Clinton WL , et al The association of team-specific workload and staffing with odds of burnout among VA primary care team members. J Gen Intern Med. 2017;32(7):760-766. doi:10.1007/s11606-017-4011-4 28233221 PMC5481228

[70] Reith TP . Burnout in United States healthcare professionals: a narrative review. Cureus. 2018;10(12):e3681. doi:10.7759/cureus.3681 PMC6367114 30761233

[71] Cetrano G Tedeschi F Rabbi L , et al How are compassion fatigue, burnout, and compassion satisfaction affected by quality of working life? Findings from a survey of mental health staff in Italy. BMC Health Serv Res. 2017;17:755. doi:10.1186/s12913-017-2726-x 29162095 PMC5696765

[72] Wagaman MA Geiger JM Shockley C Segal EA . The role of empathy in burnout, compassion satisfaction, and secondary traumatic stress among social workers. Soc Work. 2015;60(3):201-209. doi:10.1093/sw/swv014 26173361

[73] Shoji K Lesnierowska M Smoktunowicz E , et al What comes first, job burnout or secondary traumatic stress? Findings from two longitudinal studies from the US and Poland. PLoS One. 2015;10(8):e0136730. doi:10.1371/journal.pone.0136730 PMC4549333 26305222

[74] Zisook S Doran N Mortali M , et al Relationship between burnout and major depressive disorder in health professionals: a HEAR report. J Affect Disord. 2022;312:259-267. doi:10.1016/j.jad.2022.06.047 35760197

[75] Dalum HS Hem E Ekeberg Ø Reneflot A Stene-Larsen K Hauge LJ . Suicide rates among health-care professionals in Norway 1980-2021. J Affect Disord. 2024;355:399-405. doi:10.1016/j.jad.2024.03.128 38537752

[76] Chen D Ni Y Lu J Wang Y Qi Q Zhai H . Examining the impact of perceived stress, anxiety, and resilience on depression among medical staff after COVID-19 quarantine: a chain mediation analysis. Front Public Health. 2023;11:1250623. doi:10.3389/fpubh.2023.1250623 37799150 PMC10549932

[77] Najmabadi L Agénor M Tendulkar S . “Pouring from an empty cup”: manifestations, drivers, and protective factors of occupational stress among healthcare providers of trauma-informed care. J Interpers Violence. 2024;39(9-10):2041-2075. doi:10.1177/08862605231215028 38059411

[78] Dweck CS Yeager DS . A growth mindset about intelligence. In: Walton GM Crum AJ , eds. Handbook of Wise Interventions: How Social Psychology Can Help People Change. Guilford Press; 2021;9-35.

[79] Singer J Cummings C Moody SA Benuto LT . Reducing burnout, vicarious trauma, and secondary traumatic stress through investigating purpose in life in social workers. J Soc Work. 2020;20:620-638. doi:10.1177/1468017319853057

[80] VanderWeele TJ McNeely E Koh HK . Reimagining health-flourishing. JAMA. 2019;321(17):1667-1668. doi:10.1001/jama.2019.3035 30933213

[81] Seligman MEP . Positive psychology: a personal history. Annu Rev Clin Psychol. 2019;15(1):1-23. doi:10.1146/annurev-clinpsy-050718-095653 30525996

[82] Quoidbach J Mikolajczak M Gross JJ . Positive interventions: an emotion regulation perspective. Psychol Bull. 2015;141(3):655-693. doi:10.1037/a0038648 25621978

[83] Tejada-Gallardo C Blasco-Belled A Torrelles-Nadal C Alsinet C . Effects of school-based multicomponent positive psychology interventions on well-being and distress in adolescents: a systematic review and meta-analysis. J Youth Adolesc. 2020;49(10):1943-1960. doi:10.1007/s10964-020-01289-9 32683592

[84] Czyżowska N Gurba E . Does reflection on everyday events enhance meaning in life and well-being among emerging adults? Self-efficacy as mediator between meaning in life and well-being. Int J Environ Res Public Health. 2021;18:9714. doi:10.3390/ijerph18189714 34574642 PMC8472181

[85] Kovich MK Simpson VL Foli KJ Hass Z Phillips RG . Application of the PERMA model of well-being in undergraduate students. Int J Community Well-Being. 2023;6:1-20. doi:10.1007/s42413-022-00184-4 PMC9607835 36320595

[86] Bolier L Haverman M Westerhof GJ Riper H Smit F Bohlmeijer E . Positive psychology interventions: a meta-analysis of randomized controlled studies. BMC Public Health. 2013;13(1):119-138. doi:10.1186/1471-2458-13-119 23390882 PMC3599475

[87] Carayon P Cassel C Dzau VJ . Improving the system to support clinician well-being and provide better patient care. JAMA. 2019;322(22):2165-2166. doi:10.1001/jama.2019.17406 31644783

[88] Niven AS Sessler CN . Supporting professionals in critical care medicine: burnout, resiliency, and system-level change. Clin Chest Med. 2022;43(3):563-577. doi:10.1016/j.ccm.2022.05.010 36116823

[89] van Zyl LE van Vuuren HA Roll LC Stander MW . Person-environment fit and task performance: exploring the role(s) of grit as a personal resource. Curr Psychol. 2023;42:23560-23579. doi:10.1007/s12144-022-03461-9

[90] Batista JS Gondim SM . Personality and person-work environment fit: a study based on the RIASEC model. Int J Environ Res Public Health. 2022;20(1):719. doi:10.3390/ijerph20010719 36613040 PMC9819525

[91] Lanning K Wetherell G Gardiner G Weston SJ . Current research in ecological and social psychology. Curr Res Ecol Soc Psychol. 2024;6:100180. doi:10.1016/j.cresp.2024.100180

[92] Wilcox G Gibson AM Lindsay BL Prasad S Szeto ACH . Mental health in emerging adults with ADHD and/or specific learning disabilities: a scoping review. Emerg Adulthood. 2024;12(1):128-152. doi:10.1177/21676968231214837

[93] Booth R . Helping us heal: how creative life story work supports individuals and organisations to recover from trauma. J Soc Work Pract. 2022;36(1):119-127. doi:10.1080/02650533.2021.2025349

[94] Lin L Zhang X Wang P . Interconnected stressors and well-being in healthcare professionals. Appl Res Qual Life. 2025;20:459-481. doi:10.1007/s11482-024-10419-5

[95] Nocon AS Roestorf A Menéndez LMG . Positive psychology in neurodiversity: an investigation of character strengths in autistic adults in the United Kingdom in a community setting. Res Autism Spectr Disord. 2022;99:102071. doi:10.1016/j.rasd.2022.102071

[96] Williams J Kumar PA . Mediating role of self-concept on character strengths and well-being among adolescents with specific learning disorder in India. Res Dev Disabil. 2023;132:104372. doi:10.1016/j.ridd.2022.104372 36423430

[97] Rodríguez-Armendariz E Vela-Romero M Galiana A . Sensory processing challenges in children with neurodevelopmental disorders and genetic conditions: an observational study. Neurosci. 2024;5(3):339-353. doi:10.3390/neurosci5030027 39483286 PMC11467969

[98] Pinho TD Manz PH DuPaul GJ Anastopoulos AD Weyandt LL . Predictors and moderators of quality of life among college students with ADHD. J Atten Disord. 2019;23(14):1736-1745. doi:10.1177/1087054717734645 28992747 PMC6209539

[99] Song P Zha M Yang Q Zhang Y Li X Rudan I . The prevalence of adult attention-deficit hyperactivity disorder: a global systematic review and meta-analysis. J Glob Health. 2021;11:04009. doi:10.7189/jogh.11.04009 PMC7916320 33692893

[100] Sedgwick-Müller JA Müller-Sedgwick U Adamou M , et al University students with attention deficit hyperactivity disorder (ADHD): a consensus statement from the UK adult ADHD network (UKAAN). BMC Psychiatry. 2022;22(1):292. doi:10.1186/s12888-022-03898-z 35459116 PMC9027028

[101] Carriba P Lorenzón N Dierssen M . Neurodevelopmental disorders: 2023 update. Free Neuropathol. 2023;4:8. doi:10.17879/freeneuropathology-2023-4701 37347033 PMC10280276

[102] Maenner MJ Warren Z Williams AR , et al Prevalence and characteristics of autism spectrum disorder among children aged 8 years—Autism and Developmental Disabilities Monitoring Network, 11 sites, United States, 2020. MMWR Surveill Summ. 2023;72(2):1-14. doi:10.15585/mmwr.ss7202a1 PMC10042614 36952288

[103] Munawar K Nair V Roy M Javed S Sadr AJ . Autobiographical memories in individuals with autism spectrum disorders: a systematic review. Curr Psychol. 2024;43:28521-28530. doi:10.1007/s12144-024-06447-x

[104] Kirchner J Ruch W Dziobek I . Brief report: character strengths in adults with autism spectrum disorder without intellectual impairment. J Autism Dev Disord. 2016;46(10):3330-3337. doi:10.1007/s10803-016-2865-7 27457365

[105] Paneth N . The contribution of epidemiology to the understanding of neurodevelopmental disabilities. Dev Med Child Neurol. 2023;65(12):1551-1556. doi:10.1111/dmcn.15633 37149891

[106] Olusanya BO Smythe T Ogbo FA Nair MKC Scher M Davis AC . Global prevalence of developmental disabilities in children and adolescents: a systematic umbrella review. Front Public Health. 2023;11:1122009. doi:10.3389/fpubh.2023.1122009 36891340 PMC9987263

[107] Maw KJ Beattie G Burns EJ . Cognitive strengths in neurodevelopmental disorders, conditions and differences: a critical review. Neuropsychologia. 2024;197:108850. doi:10.1016/j.neuropsychologia.2024.108850 38467371

[108] Hughes C, ed . Diversity intelligence, career development, and digital/virtual work. In: Diversity Intelligence: Reimagining and Changing Perspectives. Springer Nature; 2023;107-134.

[109] Dimoff JK Kelloway EK . Implementing psychological health and safety standards: lessons from the CSA Z1003. J Occup Health Psychol. 2023;28(2):123-135.

[110] Mirick RG Bridger J McCauley J . Trauma-informed practice with individuals with suicidal thoughts and behaviors. Clin Soc Work J. Published online 2024. doi:10.1007/s10615-024-00955-w

[111] de Vries N Boone A Godderis L , et al The race to retain healthcare workers: a systematic review on factors that impact retention of nurses and physicians in hospitals. Inquiry. 2023;60:1-21. doi:10.1177/00469580231159318 PMC10014988 36912131

